# Twig and Shoot Dieback of Citrus, a New Disease Caused by *Colletotrichum* Species

**DOI:** 10.3390/cells10020449

**Published:** 2021-02-20

**Authors:** Mario Riolo, Francesco Aloi, Antonella Pane, Magdalena Cara, Santa Olga Cacciola

**Affiliations:** 1 Department of Agriculture, Food and Environment, University of Catania, 95123 Catania, Italy; mario.riolo@unirc.it (M.R.); francesco.aloi@unipa.it (F.A.); apane@unict.it (A.P.); 2 Council for Agricultural Research and Agricultural Economy Analysis, Research Centre for Olive, Citrus and Tree Fruit- Rende CS (CREA- OFA), 87036 Rende, Italy; 3 Department of Agricultural Science, Mediterranean University of Reggio Calabria, 89122 Reggio Calabria, Italy; 4 Department of Agricultural, Food and Forest Sciences, University of Palermo, 90128 Palermo, Italy; 5 Department of Plant Protection, Faculty of Agriculture and Environment, Agriculture University of Tirana (AUT), 1029 Tirana, Albania

**Keywords:** *Colletotrichum gloeosporioides*, *Colletotrichum karstii*, ITS, TUB2, pathogenicity, citrus

## Abstract

(1) Background: This study was aimed at identifying the *Colletotrichum* species associated with twig and shoot dieback of citrus, a new syndrome occurring in the Mediterranean region and also reported as emerging in California. (2) Methods: Overall, 119 *Colletotrichum* isolates were characterized. They were recovered from symptomatic trees of sweet orange, mandarin and mandarin-like fruits during a survey of citrus groves in Albania and Sicily (southern Italy). (3) Results: The isolates were grouped into two distinct morphotypes. The grouping of isolates was supported by phylogenetic sequence analysis of two genetic markers, the internal transcribed spacer regions of rDNA (ITS) and β-tubulin (TUB2). The groups were identified as *Colletotrichum gloeosporioides* and *C. karstii*, respectively. The former accounted for more than 91% of isolates, while the latter was retrieved only occasionally in Sicily. Both species induced symptoms on artificially wound inoculated twigs. *C. gloeosporioides* was more aggressive than of *C. karstii.* Winds and prolonged drought were the factor predisposing to *Colletotrichum* twig and shoot dieback. (4) Conclusions: This is the first report of *C. gloeosporioides* and *C. karstii* as causal agents of twig and shoot dieback disease in the Mediterranean region and the first report of *C. gloeosporioides* as a citrus pathogen in Albania.

## 1. Introduction

Albania with around 2000 ha of citrus groves is a relatively small citrus growing country, but has an old tradition in the production of citrus, primarily represented by mandarins and prevalently concentrated in the districts of Vlorë, Berat, Elbasan, Durrës, Tiranë, Lezhë, Shkodër and Gjirokastër [1]. Italy ranks second among European citrus producer countries, with Sicily and Calabria regions (southern Italy) accounting for approximately 63 and 19% of total domestic production of oranges and 53 and 20% of production of tangerines, respectively [2]. Sicily alone accounts for around 86% of total domestic production of lemons [2]. Moreover, Italy is the leading producer country of organic citrus fruit globally, with over 35,000 ha, corresponding to about one third of the total domestic citrus growing area [3]. Over the last years in both Albania and southern Italy, outbreaks of citrus twig and shoot dieback were observed to occur in major orange and mandarin growing areas following extreme weather events whose frequency in the Mediterranean region has increased due to climate change [4]. The disease, here named twig and shoot dieback of citrus or Colletotrichum twig and shoot dieback to stress its association with pathogenic *Colletotrichum* species, was recently described as an emerging new disease of citrus in Central Valley, California, distinct from the typical anthracnose of fruit, leaves and twigs [5,6]. Symptoms of dieback caused by *Colletotrichum* included chlorosis, crown thinning, necrotic blotches on leaves, blight and frequently gummosis of apical twigs, shoot and branch dieback [6]. Two *Colletotrichum* species, *C. gloeosporioides* and *C. karstii*, were associated with *Colletotrichum* dieback in California. Overall, neither species clearly prevailed over the other and, in pathogenicity tests, both species were able to infect twigs, although not all field symptoms were reproduced [5]. In pathogenicity tests performed in California, *C. karstii* was more aggressive than *C. gloeosporioides* [5]. Symptoms similar to twig and shoot dieback described in California were reported from countries of the Mediterranean region, including Algeria, Marocco and Tunisia, but they were always associated with other symptoms typical of anthracnose on fruits and leaves [7,8,9]. In a recent survey of citrus groves in Portugal aimed at identifying the *Colletotrichum* species associated with typical anthracnose on fruits, leaves and twigs, *C. gloeosporioides*, the dominant species, was isolated preferentially from fruits and leaves while *C. karstii* was isolated preferentially from twigs and leaves, suggesting the hypothesis of differences in host-plant organ specificity between these two species [10]. Differences in host-plant organ preference between *Colletotrichum* species complex have long been known [11]. Two of the best-known examples of host and organ specificity are provided by *C. abscissum* and *C. limetticola*, both in the *C. acutaum* complex, associated to post-bloom fruit drop (PFD) of citrus and Key Lime Anthracnose (KLA), respectively, occurring in the Americas [12,13,14,15,16]. According to the new molecular taxonomy of the genus *Colletotrichum*, based on multi-locus phylogeny and a polyphasic approach, 25 distinct *Colletotrichum* species have been so far identified to be associated to *Citrus* and allied genera worldwide. Seven of these, including *C. aenigma*, *C. ciggaro* (syn. *C. kahawae* subsp. *ciggaro*), *C. gloeosporioides sensu stricto (s.s.)* and *C. hystricis*, within the *C. gloeosporioides* species complex, *C. catinaense* and *C. karstii*, within the *C. boninense* species complex, and *C. acutatum s.s.,* within the *C. acutatum* species complex, have been reported from Italy [9,10,17,18,19,20,21,22,23,24,25,26,27,28], while in the literature there is no record of *Colletotrichum* infecting citrus from Albania. The objectives of this study were to identify the *Colletotrichum* species associated with the new disease twig and shoot dieback or Colletotrichum twig and shoot dieback of citrus in Albania and Sicily and evaluate the pathogenicity of these *Colletotrichum* species on different plant organs, including fruit, leaves and shoots.

## 2. Materials and Methods

### 2.1. Sampling and Isolation

During 2017 and 2018, citrus orchards were surveyed for twig and shoot dieback in six municipalities (Augusta, Lentini, Mineo, Misterbianco, Ramacca and Scordia) of the provinces of Catania and Syracuse (Sicily, southern Italy), and in the municipality of Xarrë, prefecture of Vlorë (Vlorë, Albania). *Colletotrichum* isolates were obtained from twigs with symptoms of blight and gumming and from leaves showing necrotic patches and mesophyll collapse. Overall, samples were collected from 10 citrus groves in Albania and six citrus groves (one for each municipality) in Sicily and from diverse citrus species and cultivars, including Clementine mandarin (*Citrus x clementina*), three cultivars of sweet orange (*Citrus × sinensis*), “Tarocco Lempso”, ‘Tarocco Scirè’ and “Lane Late”, and two mandarin-like hybrids, “Fortune” (*C.* × *clementina* × “Orlando” tangelo) and “Mandared” (*C. × clementina* “Nules” × *C. × sinensis* “Tarocco”).

To obtain fungal isolates, twig and leaf fragments (5 mm) were washed with tap water, surface sterilised with a sodium hypochlorite solution (10%) for 1 min, immersed in 70% ethanol for 30 s and rinsed in sterile distilled water (s.d.w.). After disinfection, the fragments were blotted dry on sterilised filter paper, placed in Petri dishes on potato-dextrose-agar (PDA; Oxoid Ltd., Basingstoke, UK) amended with 150 μg/mL streptomycin and incubated at 25 °C until typical *Colletotrichum* colonies were observed. Sporulating conidiomata from subcultures were collected, crushed in a drop of s.d.w. and spread in Petri dishes over the surface of PDA amended with streptomycin to obtain single-conidium isolates. After 24 h incubation at 25 °C, germinating conidia were individually transferred to Petri dishes onto PDA. Stock cultures of single-conidium isolates were mantained on PDA slants under mineral oil at 5–10 °C in the culture collection of the Molecular Plant Pathology laboratory of the Department of Agriculture, Food and Environment of the University of Catania, Italy.

### 2.2. Fungal Isolates

A total of 119 single-conidium isolates of *Colletotrichum* from citrus, representing a population of mass isolates around ten-fold larger, was characterised in this study (Table 1).

Mass isolates were obtained from symptomatic twigs and leaves and were preliminarily separated into two groups on the basis of morphotype, i.e., colony morphology, radial growth rate on PDA at 25 °C and microscopical traits, such as the shape and size of conidia and the presence of setae. About 10% of mass isolates of each morphotype and from each site were selected randomly and a single-conidium isolate was obtained from each selected mass isolate. A *C. acutatum s.s.* isolate (UWS 149) from olive (*Olea europaea*) sourced in Australia and a *C. gloeosporioides s.s.* isolate (C2) from lemon (*Citrus* × *limon*), sourced in Calabria [29], as well as a *C. karstii* (CAM) from *Camellia* sp., sourced in Sicily [26], were used as references in growth and pathogenicity tests.

### 2.3. Morphological Characteristics and Optimum Growth Temperature of Isolates

Agar plugs (5-mm-diam) were taken from the edge of actively growing cultures on PDA and transferred to the centre of 9-cm diameter Petri dishes containing PDA. Dishes were incubated at 25 °C either in the dark for 10 d to determine both the colony morphology and radial growth rate or with continuous fluorescent light to observe microscopical morphological traits. Conidial and mycelial suspensions were prepared in s.d.w. from 10-day-old cultures and examined microscopically.

Optimum temperature for radial growth was determined only for a restricted number of isolates of the two morphotypes and also for isolates of *C. acutatum* (UWS 149), *C. gloeosporioides* (C2) and *C. karstii* (CAM) used as references. Agar plugs (5-mm-diam) were taken from the edge of actively growing cultures on PDA and transferred to the centre of 9-cm diameter Petri dishes containing PDA. Dishes were incubated at 20, 25 and 30 °C in the dark (three replicates per each temperature and per each isolate). Two orthogonal diameters were measured per each colony after 3 and 7 d incubation. The experiment was repeated once with similar results, so results of only one experiment are reported.

### 2.4. DNA Extraction, PCR Amplification and Sequencing

Genomic DNA was extracted from single-conidium *Colletotrichum* isolates using the method described in Cacciola et al. [30]. The ITS1–58S–ITS2 region and the fragment of the β-tubulin 2 gene (TUB2) between exons 2 and 6 were amplified and sequenced from a complete panel of isolates as described in a previous paper [30]. Amplified products were analyzed by electrophoresis and single bands of the expected size were purified with the QIAquick PCR Purification Kit (Qiagen, Hilden, Germany) and sequenced with both forward and reverse primers by Macrogen Europe (Amsterdam, The Netherlands). The ChromasPro v. 1.5 software [31] was used to evaluate the reliability of sequences and to create consensus sequences. Unreliable sequences in which both forward and reverse sequences, or one or the other, were not successful or contained doubtful bases were re-sequenced. The ITS and TUB2 sequences obtained in the present study were deposited in GenBank and the relative accession numbers are reported in Table 1. Validated sequences representative of all species identified within the *C. boninense* and *C. gloeosporioides* species complexes were phylogenetically analysed to determine the relationship between different isolates and define their taxonomic status. Sequences from ex-type or authentic culture were included in the analysis as a reference (Table 2).

Phylogenetic analysis was conducted for the ITS and TUB2 sequences, as well as for the combined data set of the two markers using maximum likelihood and Bayesian methods. TOPALi v2 [37] was used to determine the substitution model that best fitted the data. The model HKY + I + G was selected for the Bayesian and maximum likelihood phylogenetic analysis using MrBayes v. 3.1.1 and PhyML v. 2.4.5, respectively, implemented in TOPALi. Bayesian analysis was performed with four runs conducted simultaneously for 500,000 generations with 10% sampling frequency and burn-in of 30%. Maximum likelihood was performed with 100 bootstrap replicates.

### 2.5. Pathogenicity Test

The pathogenicity of all 119 single-conidium isolates of *Colletotrichum* from citrus and the reference isolates of *C. acutatum*, *C. gloeosporioides* and *C. karstii* was tested on mature apple fruits (*Malus domestica*) ‘Fuji’ and ‘Cripps Pink’. The assay on apples was included in this study as it was often used as a standard method to evaluate the pathogenicity of *Colletotrichum* spp. from various host-plants [38]. Apples (three apples per fungal isolate) were wound inoculated by removing aseptically a piece of tissue (3 mm side) using a scalpel, then inserting a mycelim plug of the same size upside down into the pulp of the fruit and putting back in place the piece of tissue. A sterile agar plug was inserted into control apples. Apples were incubated at 25 °C and the area of the external lesion was measured seven days post inoculation (d.p.i.). In a preliminary test using a set of isolates, including the reference isolates of *C. acutatum* (UW 149), *C. gloeosporioides* (C2) and *C. karstii* (CAM), no difference in susceptibility was observed between ‘Fuji’ and ‘Cripps Pink’ apples, so in subsequent tests of the 119 *Colletotrichum* isolates sourced from citrus during the survey and the reference isolates of the three *Colletotrichum* species fruits of the two apple cultivars were used indifferently depending on the availability.

The pathogenicity of a more restricted subset of isolates, including four *C. gloeosporioides* isolates from citrus (one, Citrus ctrl 1, sourced in Albania, and three, AC5 and AC24 from twigs and AC38 from leaf, sourced in Italy) two *C. karstii* isolates (ALL2I and ALL2S sourced in Italy) and the reference isolates of *C. acutatum* from olive (UWS 149), *C. gloeosporioides* (C2) from lemon and *C. karstii* (CAM) from camellia, was tested on different citrus plant organs, including young twigs of sweet orange ‘Tarocco Scirè’, lemon (*C.* × *limon*) ‘Femminello 2Kr’ and bergamot (*C.* × *bergamia*) ‘Fantastico’, young and mature expanded leaves of sweet orange ‘Moro’ and ‘Navelina’, mature fruit of sweet orange ‘Tarocco Meli’ and lemon ‘Femminello 2Kr’ as well as green fruitlets of lemon ‘Femminello 2Kr’.

On 1 June 2020, twigs (around 0.5 cm diameter) were wound inoculated using a scalpel to lift a strip of bark and insert under the bark, upside down and in contact with the cambium, a mycelium plug (3 mm side) taken from the edge of an actively growing culture on PDA. A plug of sterile agar was inserted under the bark of control twigs. The wounds were sealed tightly with Parafilm^®^. Inoculations were carried out on four-year-old trees in an experimental field in the municipality of Mineo. Six twigs on each tree were inoculated and six served as a control. The length of necrotic lesions was recorded at 14 d.p.i. The experimental design was a complete randomized block with three replicates (trees) per each citrus variety and *Colletotrichum* isolate combination. The experiment was repeated on June 30th and the data of the two experiments were analyzed separately.

The same *Colletotrichum* isolates were used to inoculate fruits and leaves, to test their ability to produce symptoms of anthracnose. Ripe sweet orange and lemon fruits were surface disinfected with 70% ethanol, rinsed with s.d.w., blotted dry and inoculated by wounding and without wounding. Unwounded fruits were inoculated by putting mycelium plugs (3 mm side) directly on the peel (two plugs on the upper side of each fruit placed horizontally, four cm apart from each other). Control fruits received sterile agar plugs. The same number of fruits was inoculated using a scalpel to remove aseptically two small pieces (3 mm side) of peel, four cm apart from each other. Mycelium plugs of the same size were inserted upside down into the albedo and the peel pieces were replaced to cover the wounds. Sterile agar plugs were inserted into the albedo of control fruits. After inoculation, fruits were placed in humid chambers (plastic boxes with air-tight lid) on plastic rings to avoid direct contact with the humid paper and incubated at 25 °C under 16/8 h light/dark photoperiod and 90% RH. Symptoms were recorded three, six and 12 d.p.i. Four replicated fruits for each inoculation method (wounding and without wounding) and *Colletotrichum* isolate combination were included in each of the two separate experiments, one with mature sweet orange fruit and the other with mature lemon fruit, respectively. In a separate trial the same *Colletotrichum* isolates were inoculated on unripe fruitlets of lemon ‘Feminello 2Kr’. Differently from tests on mature fruit, each fruitlet was inoculated with a single plug instead of two. Symptoms were recorded three and six d.p.i.

Expanded young (from summer vegetative flushing) and mature (from spring or previous year vegetative flushing) leaves of sweet orange ‘Moro’ and ‘Navelina’ were collected on 2 October 2020 in the same citrus orchard, surface disinfected with 70% ethanol, washed with s.d.w., blotted dry and transferred to a humid chamber (plastic boxes with air-tight lid) on blotting paper soaked with s.d.w. and covered with aluminium foil to avoid direct contact between leaves and water. Leaves were inoculated on the abaxial side by wounding and without wounding. A razor blade was used to gently scrape the surface of the leaf lamina so as to create small (2 mm side) superficial lesions (six lesions per leaf, three on each side of the midrib). A mycelium plug (3 mm side) taken from the edge of an actively growing culture on PDA was put on each lesion with the side covered by mycelium in contact with the leaf surface. Six mycelium plugs (three on each side of the midrib) were placed on the lamina of unwounded leaves. Sterile agar plugs were placed on both wounded and unwounded leaves used as controls. Leaves were incubated in humid chamber at 25 °C under 16/8 h light/dark photoperiod and 90% RH and symptoms were recorded at five d.p.i. The experimental design was a complete randomized block with four replicates (leaves) for each citrus variety, type of leaf (young or mature), inoculation method (wounded or unwounded) and *Colletotrichum* isolate combination.

*Colletotrichum* isolates used in pathogenicity tests were re-isolated from the lesions and identified based on colony morphology to fulfil Koch’s postulates. The identity of the isolates obtained from artificially inoculated symptomatic twigs, fruit and leaves was also confirmed by sequencing the ITS and TUB2 regions.

### 2.6. Statistical Analysis

Data from pathogenicity tests were analyzed using RStudio v.1.2.5 (R) [39]. The means of surface areas of necrotic lesions induced by different *Colletotrichum* isolates were compared and analyzed by one-way Analysis of Variance (ANOVA) coupled with Tukey-Kramer Honestly Significant Difference (HSD) test. Likewise, ANOVA and Tukey-Kramer Honestly Significant Difference (HSD) test were applied for statistical analysis of the differences between mean colony diameters in radial growth tests of isolates at different temperatures. When comparing independent groups, Student’s t-test was used. Levene’s test was used to determine the homogeneity of variance between independent trials. No heterogeneity was detected and data from independent trials were combined.

## 3. Results

**Figure 1:**
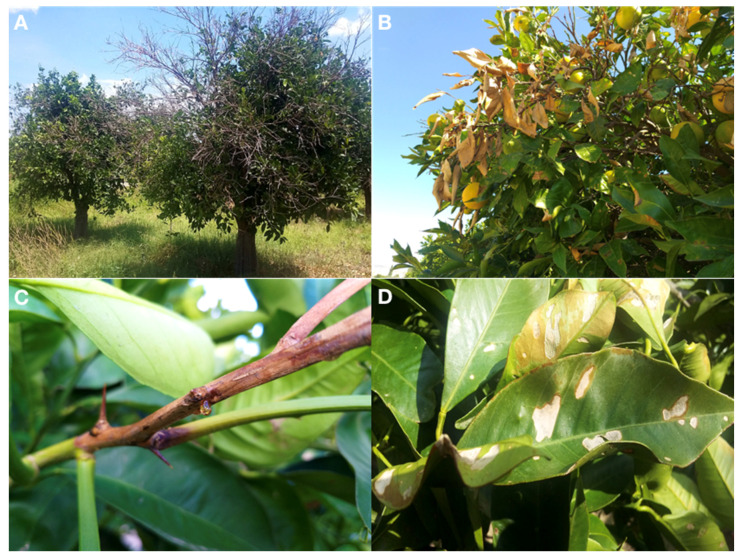
(**A**). Shoot blight and dieback of entire branches on Sweet orange ‘Tarocco Scirè’ injured by wind. (**B**) Symptoms of shoot blight incited by *Colletotrichum* infections on Sweet orange ‘Tarocco Scirè’ injured by wind. (**C**) Gumming associated with citrus shoot blight on Sweet orange ‘Tarocco Scirè’. (**D**) Leaf mesophyll collapse and necrosis caused by wind on leaves of ‘Tarocco Scirè’ sweet orange. Conidiomata of *Colletotrichum* are visible on necrotic lesions as black pin point-dots.

In all surveyed orchards, outbreaks of Colletotrichum twig and shoot dieback of citrus were observed from April to October on both young and mature trees and always following strong winds, occurring on trees suffering because of water stress after prolonged drought. Symptoms included blight and gumming of twigs (Figure 1B,C), defoliation and crown thinning as well as necrotic blotches, russeting of the abaxial side and mesophyll collapse of leaves remained still attached to the twigs (Figure 1D). In most severe cases, dieback of entire branches (Figure 1A) and death of young (one- to three-year-old) trees were observed. The incidence of the disease in a single orchard varied greatly irrespective of the age of the trees. In most orchards, affected trees were scattered and only a few twigs or shoots were symptomatic within a tree while in a few orchards more than 80% of trees were more or less seriously affected.

Symptoms were often severe on the top of the canopy of mature trees and on trees exposed to wind and suffering because of drought. Overall, about 1200 *Colletotrichum* isolates were sourced in Sicily, 93% from twigs and 7% from leaves. About 180 isolates were sourced in Albania, all from twigs. All the isolates from Albania showed the same morphology.

**Figure 2:**
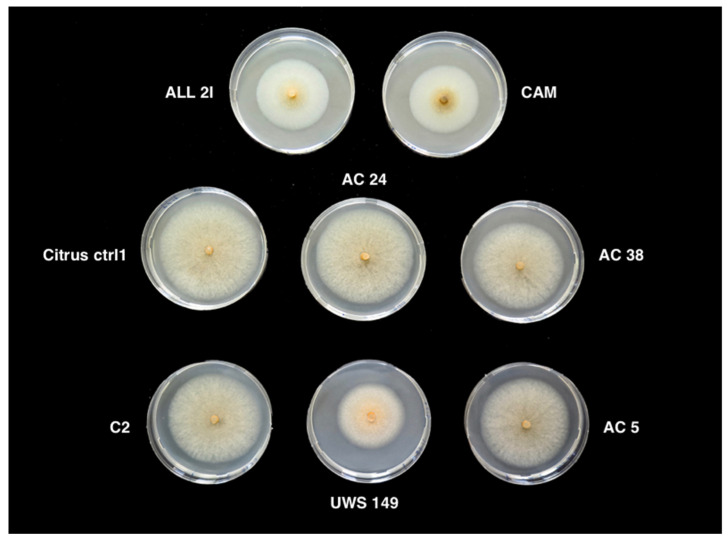
Morphology of 6-day-old colonies of *Colletotrichum gloeosporioides* (AC24, AC38, AC5, C2 and Citrus ctrl1), *C. acutatum* (UWS 149) and *C. karstii* (ALL 2I and CAM) grown on potato-dextrose-agar at 25 °C in the dark.

Colonies of these isolates on PDA were low-convex, fast-growing (10–11 mm average growth per day at 25 °C), with entire margin and dense, cottony, aerial mycelium, initially white turning to pale grey and salmon pink conidial mucilaginous masses in the centre of the colony, dark acervuli scattered over all the surface in old colonies. Colony reverse was pale orange to uniformly grey. Single-celled conidia were, hyaline, smooth, cylindrical with both ends rounded; the range of their dimensions was 11–15 × 4–6 μm. Setae were common in most isolates. Conversely, *Colletotrichum* isolates from Italy were separated into two clearly distinct groups on the basis of morphotype (Figure 2). The first group, encompassing the majority of isolates and including isolates from both twigs and leaves, showed the same morphotype as isolates from Albania. The second group, encompassing about 100 mass isolates from twigs sourced from sweet orange ‘Lane Late’ in the municipality of Augusta, showed a different morphotype. Colonies on PDA were less fast growing (8 mm average growth per day at 25 °C) and flat, with entire margin, mycelium appressed and moderately dense, white-orange to pale gray-orange, minute salmon orange conidium masses scattered over all the surfaces. The colony reverse was pale orange. Conidia were single-celled, hyaline, smooth, cylindrical with both ends rounded; the range of their dimensions was 10–17 × 4–6 μm. Setae were exceedingly rare or absent in most isolates.

**Figure 3:**
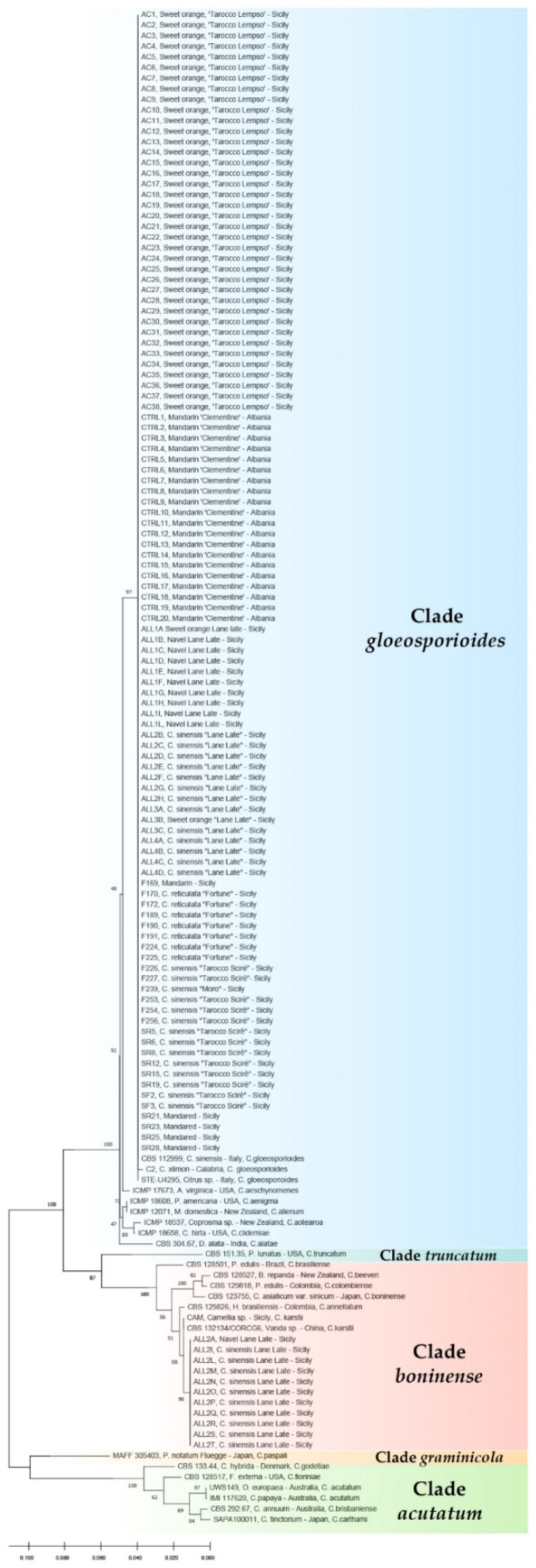
Phylogenetic tree obtained using combined internal transcribed spacers (ITS) and β-tubulin (TUB2) sequences of isolates of *Colletotrichum* spp. collected in the present study along with reference isolates of *C. karstii*, *C. gloeospoiroides*, and other representative species of the other *Colletotrichum boninense, C. gloeosporioides* and *C. acutatum* species complex. The evolutionary history was inferred using the maximum likelihood method based on the Tamura–Nei model and the tree with the highest log likelihood is shown. The percentage of trees in which the associated taxa clustered together is shown next to the branches.

The phylogenetic analysis of the combined data set of sequences from ITS and TUB2 regions of all single-conidium *Colletotrichum* isolates from citrus sourced in Albania and southern Italy (Table 1), along with sequences of the isolates of *C. acutatum* (UWS 149), *C. gloeosporioides* (C2) and *C. karstii* (CAM) used as references and the reference sequences of *Colletotrichum* species separated within the *C. gloeosporioides* and *C. boninense* species complexes, produced a phylogenetic tree (Figure 3) with a similar topology and high concordance with those reported by the authors who revised the systematics of these species complexes using multigene sequence analysis [14,22,23].

All the isolates from Albania and the isolates from southern Italy with the same morphotype were identified as *C. gloeosporioides* because they clustered (bootstrap values 100%) with the ex-type isolate of this species. Conversely, the 10 single-conidium isolates from the municipality of Augusta showing a distinct morphotype clustered (bootstrap values 100%) with ITS and TUB2 region sequences of reference isolates of *C. karstii*, including the culture from holotype isolate CBS 132134/CORCG6 and the isolate CAM from camellia sourced in Sicily. Sequences of isolates of both *C. gloeosporioides* and *C. karstii* were clearly distinct from reference sequences of *C. acutatum*.

All selected isolates of both *C. gloeosporioides* and *C. karstii* as well as the reference isolate of *C. acutatum* grew faster at 25 than at 30 and 35 °C. However, at 30 and 35 °C the *C. gloeosporioides* isolates were less inhibited than isolates of the other two species, indicating they were more thermofilic. In particular, the radial growth of *C. gloeosporioides* isolates at 30 °C was reduced only by 7 to 11% compared to the growth at 25 °C, and by 16 to 30% at 35 °C. Conversely, the growth of *C. karstii* isolates was reduced by 55 to 60% at 30 °C and by 75 up to 79 % at 35 °C. The growth of the reference isolate of *C. acutatum* was dramatically reduced at 30 and 35 °C, by 79 and 88% respectively (Table 3).

**Figure 4:**
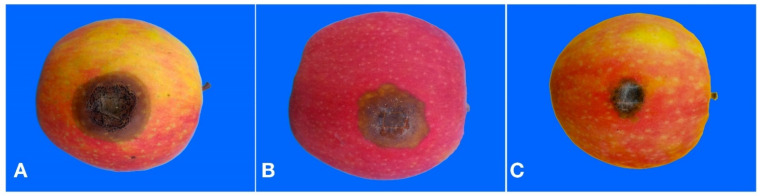
(**A**) Necrotic lesion induced by a *Colletotrichum gloeosporioides* isolate on a wound inoculated apple seven d.p.i. (**B**) Necrotic lesion induced by the *Colletotrichum acutatum* reference isolate on a wound inoculated apple seven d.p.i. (**C**) Necrotic lesion induced by a *Colletotrichum karstii* isolate on a wound inoculated apple seven d.p.i.

In pathogenicity tests on apples, all isolates were pathogenic. However, symptoms induced on apples by isolates of the three tested *Colletotrichum* species were different. Necrotic lesions induced by the *C. gloeosporioides* isolates were dark brown, with a definite margin and black conidiomata emerging from the surface in concentric rings (Figure 4A).

**Figure 5:**
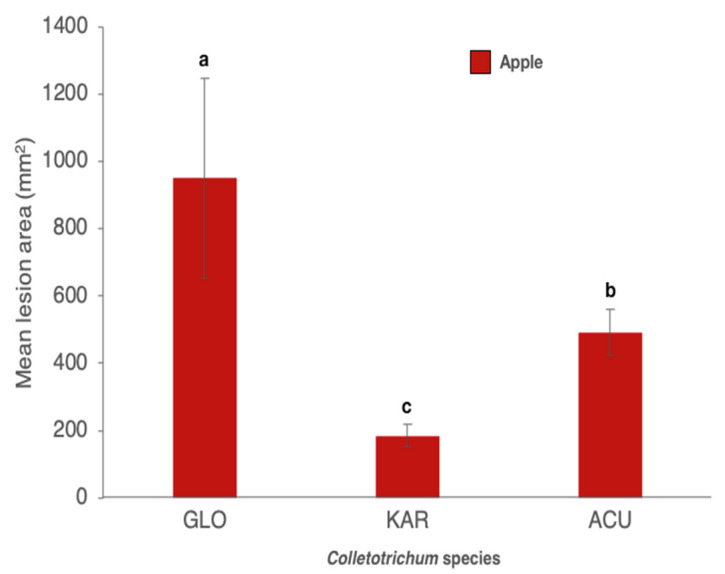
Mean area (± SD) of necrotic lesions (mm^2^) induced by *Colletotrichum gloeosporioides* (GLO), *C. karstii* (KAR) and *C. acutatum* (ACU) isolates on wound inoculated apples, seven d.p.i. Values sharing same letters are not statistically different according to Tukey’s honestly significant difference (HSD) test (*p* ≤ 0.05).

Lesions induced by the C. *acutatum* isolate were pale brown with an irregular margin, a faint white aerial mycelium around the inoculation wound and point-like orange masses of conidia scattered over the surface of the lesion (Figure 4B). Lesions on apples inoculated with *C. karstii* isolates were restricted, dark brown with a faint white mycelim emerging from the wound and without sign of sporulation (Figure 4C). No significant difference in virulence was found between isolates of the same species irrespective of their origin, so data of all isolates belonging to the same species were pooled together. *C. gloeosporioides* isolates and the reference isolate of *C. acutatum* were significantly more virulent than isolates of *C. karstii* (Figure 5).

**Figure 6:**
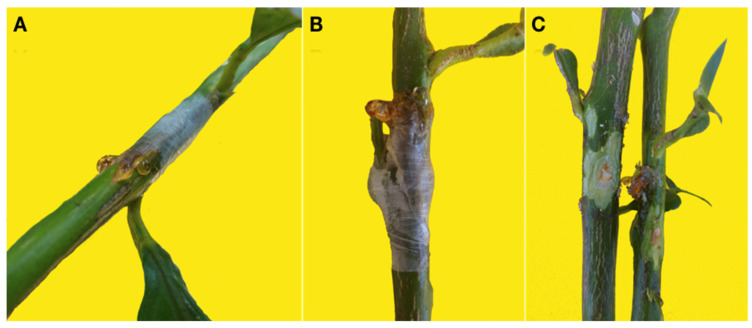
(**A**) Gumming on a twig of lemon ‘Femminello 2Kr’ wound inoculated with a *Colletotrichum gloeosporioides* isolate 14 d.p.i. (**B**) Gumming on a twig of bergamot ‘Fantastico’ wound inoculated with a *Colletotrichum gloeosporioides* isolate 14 d.p.i. (**C**) Twigs of bergamot ‘Fantastico’ wound inoculated with *Colletotrichum karstii* (left) and *C. gloeosprioides* (right) 14 d.p.i. The bark was removed to show the internal symptoms: the twig on the left shows a gum impregnation of the young xylem and a cicatricial tissue around the inoculation point while the twig on the right shows a profuse gumming extending beyond the inoculation point.

In in-field tests on twigs of sweet orange, lemon and bergamot the isolates of *C. gloeosporioides* and *C. acutatum* were more aggressive than isolates of *C. karstii*. Isolates of *C. acutatum* and *C. gloeosporioides* induced gumming in twigs of all three citrus species while isolates of *C. karstii* induced gumming only in bergamot twigs (Figure 6A–C).

**Figure 7:**
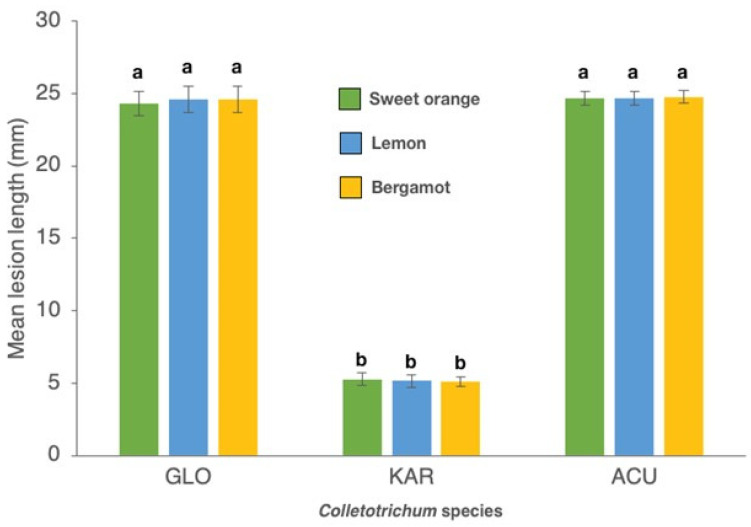
Mean length (mm) of necrotic lesions of six twigs per each of three replicated trees (±SD) induced by isolates of *Colletotrichum gloeosporioides* (GLO) (five isolates, means of 90 replicates), *C. karstii* (KAR) (three isolates, means of 54 replicates) and *C. acutatum*(ACU) (one isolate, means of 18 replicates) on in-field artificially inoculated twigs of sweet orange ‘Tarocco Scirè VCR’, lemon ‘Femminello 2Kr’ and bergamot ‘Fantastico’, 14 d.p.i. Values sharing same letters are not statistically different according to Tukey’s honestly significant difference (HSD) test (*p* ≤ 0.05).

Mean length of necrotic lesion of the bark induced by the *C. gloeosporioides* and *C. acutatum* isolates was significantly higher than the length of lesions induced by the *C. karstii* isolates (Figure 7). In twigs inoculated with *C. karstii* isolates, the necrotic lesion was localized around the inoculation point and the wound healed rapidly. No significant difference in lesion size was observed between sweet orange, lemon and bergamot twigs. Likewise, no significant difference in virulence, as determined on the basis of the size of the necrotic lesion, was observed between isolates of the same *Colletotrichum* species so data of all isolates belonging to the same species were pooled together. On control twigs, inoculation wounds healed rapidly without any symptoms of necrosis or gummosis.

**Figure 8:**
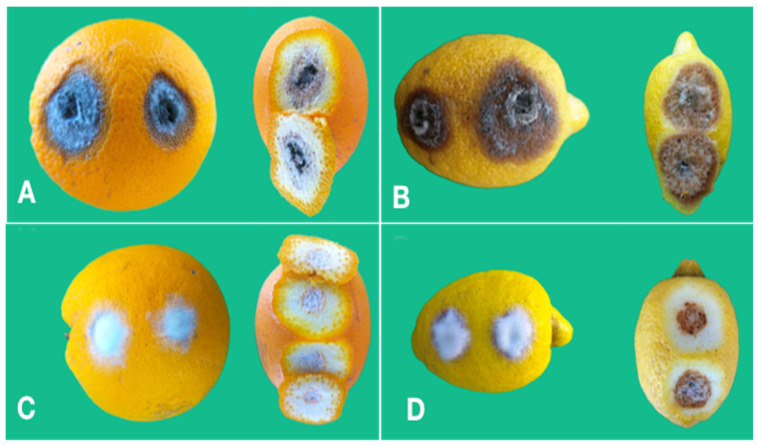
(**A**) Necrotic lesions around the inoculation point in a fruit of sweet orange ‘Tarocco Meli’ wound inoculated with an isolate of *Colletotrichum gloeosporioides* 12 d.p.i.; the peel has been removed to show that the necrosis extends into the albedo. (**B**) Necrotic lesions around the inoculation point in a fruit of lemon ‘Femminello 2Kr’ wound inoculated with an isolate of *Colletotrichum gloeosporioides* 12 d.p.i.; the peel has been removed to show that the necrosis extends into the albedo. (**C**) Aerial mycelium developed on the two inoculation points in a fruit of sweet orange ‘Tarocco Meli’ wound inoculated with the reference isolate of *Colletotrichum acutatum* 12 d.p.i.; the peel has been removed to show the necrotic lesion extending into the albedo lined by an orange coloured cicatricial tissue. (**D**). Aerial mycelium growing on the two inoculation points in a fruit of lemon ‘Femminello 2Kr’ wound inoculated with the reference isolate of *Colletotrichum acutatum* 12 d.p.i.; the peel has been removed to show the necrotic lesion extending into the albedo.

**Figure 9:**
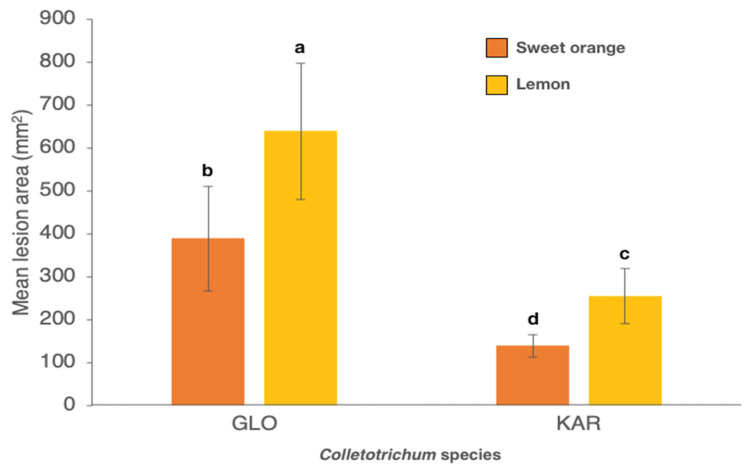
Mean area (mm^2^) of necrotic lesions (± SD) induced by isolates of *Colletotrichum gloeosporioides* ^b^ (GLO) (five isolates) and *C. karstii* ^c^ (KAR) (three isolates) ^a^ on wound inoculated fruits of sweet orange ‘Tarocco Meli’ and lemon ‘Femminello 2Kr’, 12 d.p.i. Values sharing same letters are not statistically different according to Tukey’s honestly significant difference (HSD) test (*p* ≤ 0.05). ^a^ Four fruits for each citrus species and two inoculation wounds per fruit. ^b^ Means of 40 replicates. ^c^ Means of 24 replicates.

In tests on green and mature fruit as well as on tender and mature leaves *Colletotrichum* isolates were able to induce lesions only after wounding. In pathogenicity tests on mature fruit of sweet orange and lemon, *C. gloeosporioides* isolates were more aggressive than isolates of *C. karstii* even on fruits. Isolates of both species induced a brown necrotic halo around the inoculation wound and the necrosis extended deep into the albedo (Figure 8A,B), but the mean size of lesions induced by the *C. gloeosporioides* isolates was greater than the mean size of lesions induced by the *C. karstii* isolates on both sweet orange and lemon (Figure 9). No significant difference in virulence, as determined based on the average size of the necrotic lesion, was observed between isolates of the same *Colletotrichum* species so data of isolates belonging to the same species were pooled together (Figure 9).

The reference isolate of *C. acutatum* induced quite peculiar symptoms on both sweet orange and lemon fruit as the inoculation wounds were covered by a white, cottony aerial mycelium, which masked the lesion. The necrosis extended into the albedo and reached its maximum extent 6 d.p.i., but did not expand further (Figure 8C,D).

As a consequence, the area of the external necrotic lesion cannot be measured and direct comparison with isolates of the other two *Colletotrichum* species was not possible in terms of virulence. No symptoms were observed on control fruits.

**Figure 10:**
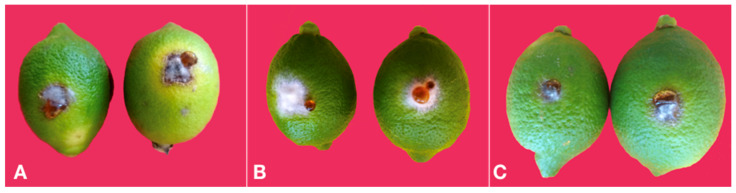
(**A**) Aerial gray mycelium and gum exudate on green fruitlets of lemon ‘Femminello 2Kr’ wound inoculated with an isolate of *Colletotrichum gloeosporioides* 6 d.p.i. (**B**) Aerial white mycelium and gum exudate on green fruitlets of lemon ‘Femminello 2Kr’ wound inoculated with the reference isolate of *Colletotrichum acutatum* 6 d.p.i. (**C**) Aerial gray mycelium and gum exudate on green fruitlets of lemon ‘Femminello 2Kr’ wound inoculated with an isolate of *Colletotrichum karstii* 6 d.p.i.

On green fruitlets of lemon differences of symptoms induced by isolates of the three *Colletotrichum* species were almost exclusively qualitative. All *C. gloeosporioides* and *C. karstii* isolates induced gumming and a very restricted necrotic lesion around the inoculation point (Figure 10A,C) while fruitlets inoculated with the *C. acutatum* isolate showed gumming and an abundant white aerial mycelium covering the lesion (Figure 10B). Symptom appeared three d.p.i. and did not evolve further over the next three days. The only symptom on control fruitlets was the necrosis of tissue plug removed temporarily to inoculate the fruitlets and replaced to plug the inoculation wound.

**Figure 11:**
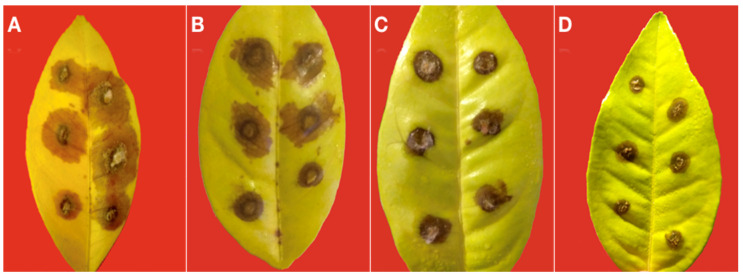
(**A**) Necrotic lesions induced by the heterologous *Colletotrichum karstii* isolate from camellia on wound inoculated young leaves of sweet orange ‘Navelina’ 5 d.p.i. (**B**) Necrotic lesions induced by the heterologous reference isolate of *Colletotrichum acutatum* from olive on wound inoculated young leaves of sweet orange ‘Navelina’ 5 d.p.i. (**C**) Necrotic lesions induced by an isolate of *Colletotrichum karstii* from citrus on wound inoculated young leaves of sweet orange ‘Moro’ 5 d.p.i. (**D**) Necrotic lesions induced by an isolate of *Colletotrichum gloeosporioides* from citrus on wound inoculated young leaves of sweet orange ‘Moro’ 5 d.p.i.

All *Colletotrichum* isolates induced circular necrotic lesions on young leaves of both ‘Navelina’ and ‘Moro’ (Figure 11A–D) while the only symptom induced on mature leaves of these two sweet orange cultivars was a translucid, very restricted dark brown halo around the inoculation point. No symptoms were observed on both young and mature control leaves.

**Figure 12:**
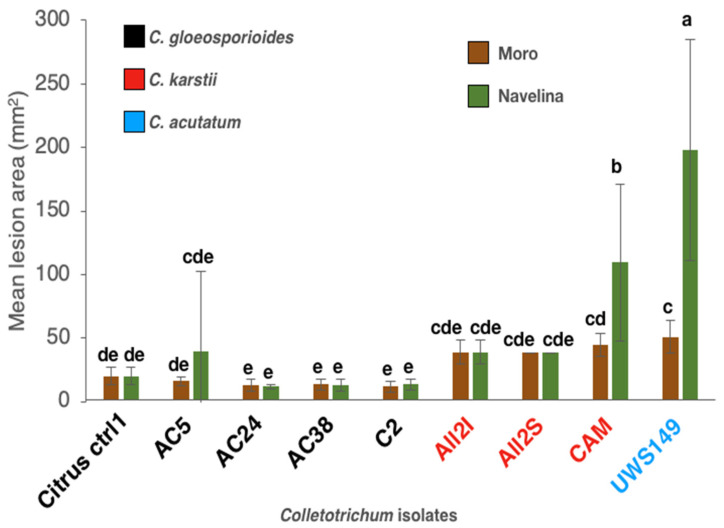
Mean area of 24 replicates (four leaves with six lesions each) of necrotic lesions (mm^2^) (± SD) incited by isolates of *C. gloeosporioides* (Citrus ctr1, AC5, AC24, AC38 and C2), *C. karstii* (All2I, All2S and CAM) and *C. acutatum* (UWS149) on wound inoculated young leaves of sweet orange ‘Moro’ and ‘Navelina’, five d.p.i. Values sharing same letters are not statistically different according to Tukey’s honestly significant difference (HSD) test (*p* ≤ 0.05).

Slight, albeit significant, differences in susceptibility were observed between ‘Moro’ and ‘Navelina’, the latter being more susceptible to the infection of aggressive isolates. There were, in fact, significant differences in virulence among the *Colletotrichum* isolates tested. Unexpectedly, the two heterologous isolates, i.e., the *C. acutatum* reference isolate from olive and the *C. karstii* reference isolate from camellia, were the most aggressive. Both the *C. karstii* isolates recovered from ‘Lane Late’ showed an intermediate virulence while the *C. gloeosporioides* isolates were slightly less virulent and did not differ significantly between each other (Figure 12).

## 4. Discussion

A new disease of citrus, named twig and shoot dieback or Colletotrichum twig and shoot dieback to stress its association with pathogenic *Colletotrichum* species, and recently observed in California, is reported for the first time from two citrus growing countries of the Mediterranean region, Albania and Italy, where it was found to be quite common and widespread. According to the modern taxonomy of *Colletotrichum* based prevalently on multilocus sequence phylogeny and consistently with the results obtained in California, the *Colletotrichum* species associated with twig and shoot dieback of citrus in Albania and Italy were identified as *C. gloeosporioides s.s.*, in the *C. gloeosporioides* species complex, and *C. karstii*, in the *C. boninense* species complex. *Colletotrichum gloeosporioides* was the only species associated with twig and shoot dieback in Albania and by far the prevalent species associated with this syndrome in Sicily. *C. gloeosporioides* is reported for the first time as a pathogen of citrus in Albania. Differently from *C. gloeosporioides*, *C. karstii* was found only sporadically, accounting for just around one third of the *Colletotrichum* isolates retrieved from a single sampling site in Sicily. The results of in-field tests provided evidence that both *Colletotrichum* species were pathogens on twigs of different citrus species, but *C. gloeosporioides* was more aggressive than *C. karstii*, indicating the former species was the main causative agent of twig and shoot dieback in surveyed areas. By contrast, in California *C. karstii* was proved to be more virulent than *C. gloeosporioides* [5]. To explain this discrepancy one can only speculate that populations of *C. gloeosporioides* and *C. karstii* associated to citrus in California are different from populations of these two species from the Mediterranean region. However, no general conclusion can be drawn as in pathogenicity tests performed in California only a single isolate of *C. gloeosporioides* and a single isolate of *C. karstii* were compared [5]. In agreement with our results, previous studies aimed at identifying the *Colletotrichum* species associated to citrus anthracnose in China and Europe, showed that among the *Colletotrichum* species recovered from citrus groves *C. gloeosporioides* was the most common and the most virulent on detached citrus fruits [17,25,27]. No significant intraspecific variability in virulence was observed among the isolates of *C. gloeosporioides* and *C. karstii* recovered from citrus in Albania and Italy. However, evidence from other studies indicate the pathogenicity of isolates of both *C. gloeosporioides* and *C. karstii* may vary even within populations originating from the same host-plant and geographic area. Marked differences in pathogenicity were reported among isolates of *C. gloeosporioides* from citrus sourced in Tunisia as well as among *C. gloeosporioides* isolates from olive sourced in Italy [19,26]. In Portugal, an isolate of *C. karstii* from sweet orange was as virulent as an isolate of *C. gloeosporioides* from lemon when inoculated on sweet orange fruits and significantly less virulent when inoculated on lemon and mandarin fruits [10]. In this study, all tested isolates of *C. gloeosporioides* were more aggressive on twigs than isolates of *C. karstii* irrespective of their origin.

*Colletotrichum gloeosporioides*, both in a broad (*C. gloeosporioides* species complex) and in a strict sense (*C. gloeosporioides s.s.*), has a wide host range and is the most common *Colletotrichum* species associated to symptoms of citrus anthracnose globally [8,17,23,24,25,40,41,42]. In this study, all isolates of *C. gloeosporioides* recovered from trees with symptom of twig and shoot dieback in Albania and Sicily were overly aggressive and were able of inducing anthracnose symptoms on fruits and leaves, as well as necrosis and gumming on twigs of different citrus species.

*C. karstii* is also a polyphagous species and among the species in the *C. boninese* complex it is the most common and the one reported from a greater number of countries and different geographical areas [14]. In Italy, it was recovered from several host-plants including olive (*Olea europaea*), and there is evidence of it as a citrus inhabitant in southern Italy since the 1990s [26,43]. In this study, isolates of *C. karstii* retrieved from citrus were less virulent than isolates of *C. gloeosporioides* on artificially inoculated fruits and twigs of both sweet orange and lemon. However, isolates of *C. karstii* from citrus demonstrated to be more virulent than *C. gloeosporioides* isolates on young leaves of sweet orange. This agrees with previous reports indicating this *Colletotrichum* species is a common foliar pathogen of citrus [10,23,24,25]. In a previous study aimed at characterizing the *Colletotrichum* species in the *C. gloeosporioides* and *C. boninense* complexes associated with olive anthracnose, *C. karstii* isolates from olive showed a low level of virulence on olive drupes, suggesting this species was an occasional pathogen on olive [26].

Overall the results of this survey do not support the hypothesis of organ specificity as a factor determining the prevalence of a *Colletotrichum* species over another in citrus trees affected by twig and shoot dieback. It is likely that the proportion of *C. gloeosporioides* and *C. karstii* isolates recovered in this study and the distribution of these two *Colletotrichum* species in surveyed areas depend on other factors conditioning their fitness and adaptative capacity. In the years following the revision of the systematics of *C. gloeosporioides* and *C. boninense* species complexes that led to the segregation of *C. karstii* as a distinct species within the *C. boninense* complex, *C. karstii* has been increasingly reported from several citrus growing countries, including China, Iran, Italy, New Zealand, Portugal, South Africa, Tunisia, Turkey and USA, always in association with other *Colletotrichum* species, mainly *C. gloeosporioides* [5,10,19,20,23,24,27,28]. This does not imply necessarily that *C. karstii* is an emerging pathogen *sensu* [4]. It seems more likely that the proliferation of reports of this *Colletotrichum* species on citrus from diverse and distant geographical areas is a consequence of the taxonomic revision of the *C. gloeosporioides* and *C. boninense* species complexes based on multilocus phylogenetic analysis, which provided a framework for a correct identification of already present cryptic species.

In the present study, a third *Colletotrichum* species included as a reference, *C. acutatum s.s.*, was shown to be as virulent as *C. gloeosporioides* on artificially inoculated twigs of different citrus species while on fruits of sweet orange and lemon induced symptoms different from the typical anthracnose. Interestingly, shoot blight is one of the symptoms of KLA, a disease caused by *C. limetticola*, also a species in the *C. acutatum* complex and affecting exclusively Key lime [14,44]. The only report of *C. acutatum s.s.* on citrus in Europe is from a small island of the Aeolian archipelago, north of Sicily, where it was recovered from leaves of lemon and sweet orange [17]. Yet this polyphagous *Colletotrichum* species, probably originating from the southern hemisphere, is already established in southern Italy on different host-plants and is replacing *C. godetiae* (syn. *C. clavatum*) as the main causal agent of olive anthracnose in Calabria [45,46,47]. In addition, there is evidence of its presence on oleander in Sicily since 2001 [29]. The threat posed by this exotic *Colletotrichum* species as a potential citrus pathogen in the Mediterranean region deserves particular attention.

The sudden outbreak of twig and shoot dieback in vast areas following winds and prolonged drought presupposes an inoculum already present and widespread throughout the citrus orchards. As a matter of fact, *Colletotrichum gloeosporioides* and *C. karstii*, like many other *Colletotrichum* species, may have different lifestyles. They may be latent pathogens, endophytes, epiphytes or saprobes and switch to a pathogenic lifestyle when host plants are under stress [48]. As stress factors have a fundamental role in triggering the infection by *Colletotrichum* species, twig and shoot dieback may be regarded as a complex disease, a definition also encompassing other emerging citrus diseases, such as dry root rot incited by species of *Fusarium s.l.* [49,50,51]. A common feature of this type of diseases is the difficulty in reproducing the syndrome in experimental conditions as even wounding may only partially substitute environmental stresses and cannot reproduce alone the effects of different stressors acting simultaneously on host-plant. This may explain the failure in reproducing all field symptoms of Colletotrichum twig and shoot dieback by artificial inoculations [5].

## 5. Conclusions

This study provided evidence that the new disease of citrus named twig and shoot dieback emerging in the Mediterranean region is caused by *Colletotrichum gloeosporioides,* and occasionally by *C. karstii*. Consistently with the results of this study, both *C. gloeosporioides* and *C. karstii* were found to be associated with the disease in Central Valley in California, but the proportion and distribution of the two *Colletotrichum* species in citrus groves of California, Albania and Italy were different. In Albania and Italy, winds and drought were identified as the stress factors predisposing the host-plant to the infections by *Colletotrichum* spp. and allowing these ubiquitous fungi to switch from a endophytic or saprophytic to a pathogenic lifestyle, while in California the predisposing stress factors have not yet been precisely determined [5,52]. A better understanding of both the diversity of *Colletotrichum* species, associated with twig and shoot dieback of citrus, and the factors triggering the outbreaks of this disease is basilar for developing effective management strategies. In California, the effectiveness of chemical treatments with fungicides, including strobilurins (azoxystrobin, pyraclostrobin and trifloxystrobin), triazoles (fenbuconazole) and copper fungicides, are being investigated as part of an integrated disease management strategy. A more sustainable management strategy, compatible with organic farming, should privilege measures aimed at both preventing or mitigating the effect of predisposing factors and reducing the amount of inoculum of *Colletotrichum* in the orchard, such as proper management of the irrigation to avoid water stress, use of windbreaks to protect the trees from winds and pruning to remove withered twigs and branches and stimulate new vegetation flushing.

## Figures and Tables

**Table 1:** Isolates of *Colletotrichum* sourced from different cultivars of *Citrus* species in Sicily (southern Italy) and Albania and characterized in this study. * Tetraploid hybrid clementine ‘Nules’x sweet orange ‘Tarocco’.

Isolate Code	Species	Host (Species and Cultivar)	Organ	Geographical Origin	Genbank Accession	
					ITS-rDNA	β-tubulin 2
AC1	*C. gloeosporioides*	Sweet orange ‘Tarocco Lempso’	twig	Ramacca (Ct)-Sicily	MT997785	MW001517
AC2	*C. gloeosporioides*	Sweet orange ‘Tarocco Lempso’	twig	Ramacca (Ct)-Sicily	MT997786	MW001518
AC3	*C. gloeosporioides*	Sweet orange ‘Tarocco Lempso’	twig	Ramacca (Ct)-Sicily	MT997787	MW001519
AC4	*C. gloeosporioides*	Sweet orange ‘Tarocco Lempso’	twig	Ramacca (Ct)-Sicily	MT997788	MW001520
AC5	*C. gloeosporioides*	Sweet orange ‘Tarocco Lempso’	twig	Ramacca (Ct)-Sicily	MT997789	MW001521
AC6	*C. gloeosporioides*	Sweet orange ‘Tarocco Lempso’	twig	Ramacca (Ct)-Sicily	MT997790	MW001522
AC7	*C. gloeosporioides*	Sweet orange ‘Tarocco Lempso’	twig	Ramacca (Ct)-Sicily	MT997791	MW001523
AC8	*C. gloeosporioides*	Sweet orange ‘Tarocco Lempso’	twig	Ramacca (Ct)-Sicily	MT997792	MW001524
AC9	*C. gloeosporioides*	Sweet orange ‘Tarocco Lempso’	twig	Ramacca (Ct)-Sicily	MT997793	MW001525
AC10	*C. gloeosporioides*	Sweet orange ‘Tarocco Lempso’	twig	Ramacca (Ct)-Sicily	MT997794	MW001526
AC11	*C. gloeosporioides*	Sweet orange ‘Tarocco Lempso’	twig	Ramacca (Ct)-Sicily	MT997795	MW001527
AC12	*C. gloeosporioides*	Sweet orange ‘Tarocco Lempso’	twig	Ramacca (Ct)-Sicily	MT997796	MW001528
AC13	*C. gloeosporioides*	Sweet orange ‘Tarocco Lempso’	twig	Ramacca (Ct)-Sicily	MT997797	MW001529
AC14	*C. gloeosporioides*	Sweet orange ‘Tarocco Lempso’	twig	Ramacca (Ct)-Sicily	MT997798	MW001530
AC15	*C. gloeosporioides*	Sweet orange ‘Tarocco Lempso’	twig	Ramacca (Ct)-Sicily	MT997799	MW001531
AC16	*C. gloeosporioides*	Sweet orange ‘Tarocco Lempso’	twig	Ramacca (Ct)-Sicily	MT997800	MW001532
AC17	*C. gloeosporioides*	Sweet orange ‘Tarocco Lempso’	twig	Ramacca (Ct)-Sicily	MT997801	MW001533
AC18	*C. gloeosporioides*	Sweet orange ‘Tarocco Lempso’	twig	Ramacca (Ct)-Sicily	MT997802	MW001534
AC19	*C. gloeosporioides*	Sweet orange ‘Tarocco Lempso’	twig	Ramacca (Ct)-Sicily	MT997803	MW001535
AC20	*C. gloeosporioides*	Sweet orange ‘Tarocco Lempso’	twig	Ramacca (Ct)-Sicily	MT997804	MW001536
AC21	*C. gloeosporioides*	Sweet orange ‘Tarocco Lempso’	twig	Ramacca (Ct)-Sicily	MT997805	MW001537
AC22	*C. gloeosporioides*	Sweet orange ‘Tarocco Lempso’	twig	Ramacca (Ct)-Sicily	MT997806	MW001538
AC23	*C. gloeosporioides*	Sweet orange ‘Tarocco Lempso’	twig	Ramacca (Ct)-Sicily	MT997807	MW001539
AC24	*C. gloeosporioides*	Sweet orange ‘Tarocco Lempso’	twig	Ramacca (Ct)-Sicily	MT997808	MW001540
AC25	*C. gloeosporioides*	Sweet orange ‘Tarocco Lempso’	twig	Ramacca (Ct)-Sicily	MT997809	MW001541
AC26	*C. gloeosporioides*	Sweet orange ‘Tarocco Lempso’	twig	Ramacca (Ct)-Sicily	MT997810	MW001542
AC27	*C. gloeosporioides*	Sweet orange ‘Tarocco Lempso’	twig	Ramacca (Ct)-Sicily	MT997811	MW001543
AC28	*C. gloeosporioides*	Sweet orange ‘Tarocco Lempso’	twig	Ramacca (Ct)-Sicily	MT997812	MW001544
AC29	*C gloeosporioides.*	Sweet orange ‘Tarocco Lempso’	twig	Ramacca (Ct)-Sicily	MT997813	MW001545
AC30	*C. gloeosporioides*	Sweet orange ‘Tarocco Lempso’	twig	Ramacca (Ct)-Sicily	MT997814	MW001546
AC31	*C. gloeosporioides*	Sweet orange ‘Tarocco Lempso’	twig	Ramacca (Ct)-Sicily	MT997815	MW001547
AC32	*C. gloeosporioides*	Sweet orange ‘Tarocco Lempso’	twig	Ramacca (Ct)-Sicily	MT997816	MW001548
AC33	*C. gloeosporioides*	Sweet orange ‘Tarocco Lempso’	twig	Ramacca (Ct)-Sicily	MT997817	MW001549
AC34	*C. gloeosporioides*	Sweet orange ‘Tarocco Lempso’	twig	Ramacca (Ct)-Sicily	MT997818	MW001550
AC35	*C. gloeosporioides*	Sweet orange ‘Tarocco Lempso’	leaf	Ramacca (Ct)-Sicily	MT997819	MW001551
AC36	*C. gloeosporioides*	Sweet orange ‘Tarocco Lempso’	leaf	Ramacca (Ct)-Sicily	MT997820	MW001552
AC37	*C. gloeosporioides*	Sweet orange ‘Tarocco Lempso’	leaf	Ramacca (Ct)-Sicily	MT997821	MW001553
AC38	*C. gloeosporioides*	Sweet orange ‘Tarocco Lempso’	leaf	Ramacca (Ct)-Sicily	MT997822	MW001554
ALL1A	*C. gloeosporioides*	Sweet orange ‘Lane late’	leaf	Augusta (Sr)-Sicily	MT997843	MW001575
ALL1B	*C. gloeosporioides*	Sweet orange ‘Lane late’	leaf	Augusta (Sr)-Sicily	MT997844	MW001576
ALL1C	*C. gloeosporioides*	Sweet orange ‘Lane late’	leaf	Augusta (Sr)-Sicily	MT997845	MW001577
ALL1D	*C. gloeosporioides*	Sweet orange ‘Lane late’	leaf	Augusta (Sr)-Sicily	MT997846	MW001578
ALL1E	*C. gloeosporioides*	Sweet orange ‘Lane late’	twig	Augusta (Sr)-Sicily	MT997847	MW001579
ALL1F	*C. gloeosporioides*	Sweet orange ‘Lane late’	twig	Augusta (Sr)-Sicily	MT997848	MW001580
ALL1G	*C. gloeosporioides*	Sweet orange ‘Lane late’	twig	Augusta (Sr)-Sicily	MT997849	MW001581
ALL1H	*C. gloeosporioides*	Sweet orange ‘Lane late’	twig	Augusta (Sr)-Sicily	MT997850	MW001582
ALL1I	*C. gloeosporioides*	Sweet orange ‘Lane late’	twig	Augusta (Sr)-Sicily	MT997851	MW001583
ALL1L	*C. gloeosporioides*	Sweet orange ‘Lane late’	twig	Augusta (Sr)-Sicily	MT997852	MW001584
ALL2A	*C.karstii*	Sweet orange ‘Lane late’	twig	Augusta (Sr)-Sicily	MT997853	MW001545
ALL2B	*C. gloeosporioides*	Sweet orange ‘Lane late’	twig	Augusta (Sr)-Sicily	MT997854	MW001585
ALL2C	*C. gloeosporioides*	Sweet orange ‘Lane late’	twig	Augusta (Sr)-Sicily	MT997855	MW001586
ALL2D	*C. gloeosporioides*	Sweet orange ‘Lane late’	twig	Augusta (Sr)-Sicily	MT997856	MW001587
ALL2E	*C. gloeosporioides*	Sweet orange ‘Lane late’	twig	Augusta (Sr)-Sicily	MT997857	MW001588
ALL2F	*C. gloeosporioides*	Sweet orange ‘Lane late’	twig	Augusta (Sr)-Sicily	MT997858	MW001589
ALL2G	*C. gloeosporioides*	Sweet orange ‘Lane late’	twig	Augusta (Sr)-Sicily	MT997859	MW001590
ALL2H	*C. gloeosporioides*	Sweet orange ‘Lane late’	twig	Augusta (Sr)-Sicily	MT997860	MW001591
ALL2I	*C. karstii*	Sweet orange ‘Lane late’	twig	Augusta (Sr)-Sicily	MT997861	MW001546
ALL2L	*C. karstii*	Sweet orange ‘Lane late’	twig	Augusta (Sr)-Sicily	MT997862	MW001547
ALL2M	*C. karstii*	Sweet orange ‘Lane late’	twig	Augusta (Sr)-Sicily	MT997863	MW001548
ALL2N	*C. karstii*	Sweet orange ‘Lane late’	twig	Augusta (Sr)-Sicily	MT997864	MW001549
ALL2O	*C. karstii*	Sweet orange ‘Lane late’	twig	Augusta (Sr)-Sicily	MT997865	MW001550
ALL2P	*C. karstii*	Sweet orange ‘Lane late’	twig	Augusta (Sr)-Sicily	MT997866	MW001551
ALL2Q	*C. karstii*	Sweet orange ‘Lane late’	twig	Augusta (Sr)-Sicily	MT997867	MW001552
ALL2R	*C. karstii*	Sweet orange ‘Lane late’	twig	Augusta (Sr)-Sicily	MT997868	MW001553
ALL2S	*C. karstii*	Sweet orange ‘Lane late’	twig	Augusta (Sr)-Sicily	MT997869	MW001554
ALL2T	*C. karstii*	Sweet orange ‘Lane late’	twig	Augusta (Sr)-Sicily	MT997870	MW001555
ALL3A	*C. gloeosporioides*	Sweet orange ‘Lane late’	twig	Augusta (Sr)-Sicily	MT997871	MW001592
ALL3B	*C. gloeosporioides*	Sweet orange ‘Lane late’	twig	Lentini (Sr)-Sicily	MT997872	MW001593
ALL3C	*C. gloeosporioides*	Sweet orange ‘Lane late’	twig	Lentini (Sr)-Sicily	MT997873	MW001594
ALL4A	*C. gloeosporioides*	Sweet orange ‘Lane late’	twig	Lentini (Sr)-Sicily	MT997874	MW001595
ALL4B	*C. gloeosporioides*	Sweet orange ‘Lane late’	twig	Lentini (Sr)-Sicily	MT997875	MW001596
ALL4C	*C. gloeosporioides*	Sweet orange ‘Lane late’	twig	Lentini (Sr)-Sicily	MT997876	MW001597
ALL4D	*C. gloeosporioides*	Sweet orange ‘Lane late’	twig	Scordia (Ct)-Sicily	MT997877	MW001598
F169	*C. gloeosporioides*	Mandarin ‘Fortune’	twig	Scordia (Ct)-Sicily	MW506960	MW513961
F170	*C. gloeosporioides*	Mandarin ‘Fortune’	twig	Scordia (Ct)-Sicily	MW506961	MW513962
F172	*C. gloeosporioides*	Mandarin ‘Fortune’	twig	Scordia (Ct)-Sicily	MW506962	MW513963
F189	*C. gloeosporioides*	Mandarin ‘Fortune’	twig	Scordia (Ct)-Sicily	MW506963	MW513964
F190	*C. gloeosporioides*	Mandarin ‘Fortune’	twig	Scordia (Ct)-Sicily	MW506964	MW513965
F191	*C. gloeosporioides*	Mandarin ‘Fortune’	twig	Misterbianco (Ct)-Sicily	MW506965	MW513966
F224	*C. gloeosporioides*	Sweet orange ‘Tarocco Scirè’	twig	Misterbianco (Ct)-Sicily	MW506966	MW513967
F225	*C. gloeosporioides*	Sweet orange ‘Tarocco Scirè’	twig	Misterbianco (Ct)-Sicily	MW506967	MW513968
F226	*C. gloeosporioides*	Sweet orange ‘Tarocco Scirè’	twig	Misterbianco (Ct)-Sicily	MW506968	MW513969
F227	*C. gloeosporioides*	Sweet orange ‘Tarocco Scirè’	twig	Misterbianco (Ct)-Sicily	MW506969	MW513970
F239	*C. gloeosporioides*	Sweet orange ‘Moro’	twig	Carlentini (Sr)-Sicily	MW506970	MW513971
F253	*C. gloeosporioides*	Sweet orange ‘Tarocco Scirè’	twig	Misterbianco (Ct)-Sicily	MW506971	MW513972
F254	*C. gloeosporioides*	Sweet orange ‘Tarocco Scirè’	twig	Misterbianco (Ct)-Sicily	MW506972	MW513973
F256	*C. gloeosporioides*	Sweet orange ‘Tarocco Scirè’	leaf	Misterbianco (Ct)-Sicily	MW506973	MW513974
SR 5	*C. gloeosporioides*	Sweet orange ‘Tarocco Scirè’	twig	Mineo (Ct)-Sicily	MW506974	MW513975
SR6	*C. gloeosporioides*	Sweet orange ‘Tarocco Scirè’	twig	Mineo (Ct)-Sicily	MW506975	MW513976
SR8	*C. gloeosporioides*	Sweet orange ‘Tarocco Scirè’	twig	Mineo (Ct)-Sicily	MW506976	MW513977
SR12	*C. gloeosporioides*	Sweet orange ‘Tarocco Scirè’	twig	Mineo (Ct)-Sicily	MW506977	MW513978
SR 15	*C. gloeosporioides*	Sweet orange ‘Tarocco Scirè’	twig	Mineo (Ct)-Sicily	MW506978	MW513979
SR 19	*C. gloeosporioides*	Sweet orange ‘Tarocco Scirè’	twig	Mineo (Ct)-Sicily	MW506979	MW513980
SF 2	*C. gloeosporioides*	Sweet orange ‘Tarocco Scirè’	leaf	Mineo (Ct)-Sicily	MW506980	MW513981
SF 3	*C. gloeosporioides*	Sweet orange ‘Tarocco Scirè’	leaf	Mineo (Ct)-Sicily	MW506981	MW513982
SR 21	*C. gloeosporioides*	Mandarin-like hybrid ‘Mandared’ *	twig	Mineo (Ct)-Sicily	MW506982	MW513983
SR 23	*C. gloeosporioides*	Mandarin-like hybrid ‘Mandared’ *	twig	Mineo (Ct)-Sicily	MW506983	MW513984
SR 25	*C. gloeosporioides*	Mandarin-like hybrid ‘Mandared’ *	twig	Mineo (Ct)-Sicily	MW506984	MW513985
SR 28	*C. gloeosporioides*	Mandarin-like hybrid ‘Mandared’ *	leaf	Mineo (Ct)-Sicily	MW506985	MW513986
Citrus Ctrl 1	*C. gloeosporioides*	Clementine mandarin	twig	Xarre-Albania	MT997823	MW001555
Citrus Ctrl 2	*C. gloeosporioides*	Clementine mandarin	twig	Xarre-Albania	MT997824	MW001556
Citrus Ctrl 3	*C. gloeosporioides*	Clementine mandarin	twig	Xarre-Albania	MT997825	MW001557
Citrus Ctrl 4	*C. gloeosporioides*	Clementine mandarin	twig	Xarre-Albania	MT997826	MW001558
Citrus Ctrl 5	*C. gloeosporioides*	Clementine mandarin	twig	Xarre-Albania	MT997827	MW001559
Citrus Ctrl 6	*C. gloeosporioides*	Clementine mandarin	twig	Xarre-Albania	MT997828	MW001560
Citrus Ctrl 7	*C. gloeosporioides*	Clementine mandarin	twig	Xarre-Albania	MT997829	MW001561
Citrus Ctrl 8	*C. gloeosporioides*	Clementine mandarin	twig	Xarre-Albania	MT997830	MW001562
Citrus Ctrl 9	*C. gloeosporioides*	Clementine mandarin	twig	Xarre-Albania	MT997831	MW001563
Citrus Ctrl 10	*C. gloeosporioides*	Clementine mandarin	twig	Xarre-Albania	MT997832	MW001564
Citrus Ctrl 11	*C. gloeosporioides*	Clementine mandarin	twig	Xarre-Albania	MT997833	MW001565
Citrus Ctr 12	*C. gloeosporioides*	Clementine mandarin	twig	Xarre-Albania	MT997834	MW001566
Citrus Ctrl 13	*C. gloeosporioides*	Clementine mandarin	twig	Xarre-Albania	MT997835	MW001567
Citrus Ctrl 14	*C. gloeosporioides*	Clementine mandarin	twig	Xarre-Albania	MT997836	MW001568
Citrus Ctrl 15	*C. gloeosporioides*	Clementine mandarin	twig	Xarre-Albania	MT997837	MW001569
Citrus Ctrl 16	*C. gloeosporioides*	Clementine mandarin	twig	Xarre-Albania	MT997838	MW001570
Citrus Ctrl 17	*C. gloeosporioides*	Clementine mandarin	twig	Xarre-Albania	MT997839	MW001571
Citrus Ctrl 18	*C. gloeosporioides*	Clementine mandarin	twig	Xarre-Albania	MT997840	MW001572
Citrus Ctrl 19	*C. gloeosporioides*	Clementine mandarin	twig	Xarre-Albania	MT997841	MW001573
Citrus Ctrl 20	*C. gloeosporioides*	Clementine mandarin	twig	Xarre-Albania	MT997842	MW001574
UWS 149	*C. acutatum*	*Olea europaea*	fruit	Agonis Ridge WA-Australia	JN121186	JN121273
C2	*C. gloeosporioides*	*Citrus x limon*	fruit	Lamezia Terme (CZ)-Calabria	JN121211	JN121298
CAM	*C. karstii*	*Camellia* sp.	leaf	Sicily	KC425664	KC425716

* holotype; ** epitype.

**Table 2:** GenBank accession numbers of sequences of the isolates of worldwide origin used as references in phylogenetic analyses.

Species	Isolate	Clade	Origin	Host	Source	GenBank Accession Number	
						ITS-rDNA	β-tubulin 2
*C. acutatum* **	IMI 117620	*acutatum*	Australia	*C.papaya*	[32]	FJ788417	FJ788419
*C. aenigma* *	ICMP 18608	*gloeosporioides*	USA	*Persea americana*	[23]	JX010244	JX010389
*C. aeschynomenes* *	ICMP 17673	*gloeosporioides*	USA	*A. virginica*	[23]	JX010176	JX010392
*C. alatae* *	CBS 304.67	*gloeosporioides*	India	*Dioscorea alata*	[23]	JX010190	JX010383
*C. alienum* *	ICMP 12071	*gloeosporioides*	New Zealand	*Malus domestica*	[23]	JX010251	JX010411
*C. annellatum* *	CBS 129826	*boninense*	Colombia	*Hevea brasiliensis*	[14]	JQ005222	JQ005656
*C. aotearoa* *	ICMP 18537	*gloeosporioides*	New Zealand	*Coprosma sp*	[23]	JX010205	JX010420
*C. beeveri* *	CBS 128527	*boninense*	New Zealand	*B. repanda*	[14]	JQ005171	JQ005605
*C. boninense* *	CBS 123755	*boninense*	Japan	*C. asiaticum var. sinicum*	[14]	JQ005153	JQ005588
*C. brasiliense* *	CBS 128501	*boninense*	Brazil	*Passiflora edulis*	[14]	JQ005235	JQ005669
*C. brisbaniense* *	CBS 292.67	*acutatum*	Australia	*C. annuum*	[22]	JQ948291	JQ949942
*C. carthami* **	SAPA100011	*acutatum*	Japan	*C. tinctorium*	[33]	AB696998	AB696992
*C. clidemiae* *	ICMP 18658	*gloeosporioides*	USA	*Clidemia hirta*	[23]	JX010265	JX010438
*C. colombiense* *	CBS 129818	*boninense*	Colombia	*Passiflora edulis*	[14]	JQ005174	JQ005608
*C. fioriniae* *	CBS 128517	*acutatum*	USA	*Fiorinia externa*	[22]	JQ948292	JQ949943
*C. gloeosporioides* **	CBS 112999	*gloeosporioides*	Italy	*Citrus sinensis*	[14]	JQ005152	JQ005587
*C. gloeosporioides* *	STE-U4295	*gloeosporioides*	Italy	*Citrus* sp.	[34]	AY376532	AY376580
*C. godetiae* *	CBS 133.44	*gloeosporioides*	Denmark	*Clarkia hybrida*	[14]	JQ948402	JQ950053
*C. karstii* *	CBS 132134/ CORCG6	*boninense*	China	*Vanda* sp.	[35]	HM585409	HM585428
*C. paspali* *	MAFF 305403	*graminicola*	Japan	*P. notatum Fluegge*	[36]	EU554100	JX519244;
*C.truncatum* **	CBS 151.35	*truncatum*	USA	*Phaseolus lunatus*	[36]	GU227862	GU228156

* ex-holotype; ** epitype.

**Table 3:** Mean radial growth rates of colonies of *Colletotrichum* spp., isolates on PDA at three different temperatures, as determined after 7 d of incubation.

*Colletotrichum* spp.	Isolate	25 °C (mm d^−1^) Mean ± S.D. ^a^	30 °C (mm d^−1^) Mean ± S.D. ^a^	35 °C (mm d^−1^) Mean ± S.D. ^a^
*C. acutatum*	UWS 14	57 ± 0.6	12 ± 0.5	8 ± 0.3
*C. gloeosporioides*	AC 24	79 ± 0.5	70 ± 0.8	64 ± 1.2
*C. gloeosporioides*	Citrus ctrl1	76 ± 0.6	70 ± 1.6	53 ± 17.6
*C. gloeosporioides*	AC 5	75 ± 0.6	69 ± 0.6	63 ± 1.8
*C. gloeosporioides*	C2	75 ± 0.8	68 ± 0.3	63 ± 0.3
*C. gloeosporioides*	AC 38	73 ± 1.1	67 ± 1.3	62 ± 0.6
*C. karstii*	ALL 2I	57 ± 5.4	26 ± 1.9	12 ± 0
*C. karstii*	CAM	56 ± 1.1	22 ± 0.8	25 ± 0.3

^a^ Mean of three replicate Petri dishes.

## Data Availability

Not pertinent.
